# Virtual reality as a tool to enhance clinical psychology students' self-knowledge and self-awareness—a proof-of-concept study

**DOI:** 10.3389/fpsyg.2025.1659873

**Published:** 2026-01-05

**Authors:** Signe Hjelen Stige, Endre Visted

**Affiliations:** Department of Clinical Psychology, University of Bergen, Bergen, Norway

**Keywords:** clinical training, presence, self-awareness, self-knowledge, simulation sickness, virtual reality

## Abstract

**Objective:**

Exploring the use of Virtual Reality (VR) as a tool to enhance self-knowledge and self-awareness among psychology students – both conceptually and empirically.

**Conceptual contribution:**

Details are provided on the development, content, and organization of a VR-based exercise focusing on emotional recognition in oneself and others.

**Empirical exploration:**

A proof-of-concept study with psychology students who had completed the VR exercise as part of their training. Forty-two out of the 94 students consented to participate and completed an online survey with open and closed questions mapping students' experiences with participating in the VR-exercise, including levels of presence and simulation sickness, and perceived benefit of the exercise in strengthening self-knowledge and self-awareness.

**Results:**

Results indicated that the VR exercise was generally well received. Qualitative data highlighted the value of VR in simulating realistic emotional scenarios, although some participants noted difficulties recognizing emotions without contextual information. Quantitative data showed that participants who reported that they generally found it easier to recognize others' emotions also tended to find it easier to recognize both their own and others' emotions during the VR exercise. The same result was found for the general tendency to recognize one's own emotions. Importantly, higher levels of presence during the VR exercise and not general ease of recognizing emotions in others was significantly correlated with perceived benefit of the exercise. Levels of presence were not significantly impacted by prior experience with VR or discomfort, but perceived ease of recognizing emotions in others was negatively associated with presence.

**Conclusion:**

The results from this proof-of-concept study highlight the potential of using VR in clinical training to offer students immersive, controlled experiences that might foster self-knowledge and moment-to-moment self-awareness. It thus points to the need for further development and research on VR as a tool in clinical training.

## Introduction

The ability to know and understand oneself and to be aware of how one is affected and reacting in a particular moment is considered vital in clinical work (e.g., American Psychological Association, 1947, 2006; Benbassat and Baumal, 2005; Jennings and Skovholt, 1999; Rodolfa et al., 2005). This capacity of therapist self-awareness can be seen as consisting of a more stable base of self-knowledge, and a more fluctuating capacity for moment-to-moment self-awareness (Williams et al., 2003). Together, self-knowledge and moment-to-moment self-awareness support clinical work, for example by making it possible for a clinician to notice when one's reactions are stronger or differs from the expected reaction in a situation (e.g., countertransference reactions), by using one's own reactions in the situation to approach the client's perspective (empathic attunement), or in making clinical decisions (e.g. therapist responsiveness or diagnostic assessment) (Williams et al., 2003; Hill, 2014; Watson and Wiseman, 2021; Williams et al., 2008).

Although self-awareness is considered vital for clinical work, it has proved difficult to agree upon ways to define and measure therapist self-awareness in meaningful ways, as well as to empirically isolate the effect of therapist self-awareness on performance or outcome (Benbassat and Baumal, 2005; Williams et al., 2003, 2008; Donati and Watts, 2005). Moreover, the impact of self-awareness on clinicians' ability to remain attuned and focused on clinical tasks is ambiguous. Research suggests that increased self-focused attention can heighten emotional intensity, which may, in turn, interfere with the execution of clinical tasks. This lead Williams et al. (2003, 2008) to differentiate between hindering and helpful self-awareness. The importance of being able to quickly switch perspectives and integrate information from several sources of information in clinical work also points to the importance of cognitive flexibility and presence in helpful moment-to-moment self-awareness, and in clinical work more generally (e.g., Watson and Wiseman, 2021).

The complexity briefly outlined so far points to the importance of clearly stating how therapist self-awareness is defined. In this article we understand therapist self-awareness in line with Williams et al. (2003), as consisting of self-knowledge and moment-to-moment self-awareness. However, when discussing moment-to-moment self-awareness, it is not limited to hindering self-awareness. We teach at a 6-year integrated program in clinical psychology. Much of the literature will therefore be drawn from the field of psychotherapy, and we use the term therapist self-awareness. We believe, however, that a focus on clinician self-awareness is relevant across health disciplines.

Given the importance of therapist self-awareness for clinical work, different paths to enhance self-awareness in students have been explored, both involving a broader focus on personal development, for example through experiential groups, self-reflection, or personal therapy, and a narrower focus on self-awareness training (Grimmer and Tribe, 2001; Knapp et al., 2017a,b; Luke and Kiweewa, 2010; Payne, 1999; Topuz and Arasan, 2014). Moreover, one can distinguish between direct and indirect programs to promote students' clinically relevant self-knowledge (e.g., Benbassat and Baumal, 2005), where direct approaches typically involve classroom instruction and group discussions, while indirect approaches mainly focus on teaching clinical skills, but where experience from carrying out the task in combination with feedback on performance is thought to provide opportunities for self-reflection on part of the student.

Currently there is, therefore, no established way of supporting students' development of therapist self-awareness through clinical training. While the more direct approaches reduce cognitive load on students, by potentially allowing room for reflection and experiential work without having to learn new clinical skills at the same time, the challenge is to find ways to engage students sufficiently in activities, so they are not merely passive observers. Additionally, although personal therapy and experiential groups have great potential to facilitate self-knowledge, making these activities mandatory raises ethical concerns (Atkinson, 2006). Given the more hindering aspects of moment-to-moment self-awareness there might also be a need for low-risk arenas to strengthen this capacity, especially when students are first learning new clinical skills, as opposed to real clinical encounters. We therefore need to find ways to incorporate learning activities that provide students with the opportunity to practice key clinical skills, including self-knowledge and heightened moment-to-moment self-awareness in a safe context without the risk of inflicting harm on patients. Such activities would form the foundation to build more complex clinical skills, including the ability to shift between perspectives (e.g., between their own and their patients') to integrate information from different sources in clinical decision making (e.g., Watson, 2002). In this context it is interesting to explore how new technology can support these learning processes in clinical training.

Virtual reality (VR) offers a unique opportunity to enhance the development of self-knowledge and moment-to-moment self-awareness in clinical psychology students. One of its key advantages is the ability to immerse students in realistic, simulated clinical environments that are controlled, providing a low risk setting for practice and learning. Virtual environments are typically delivered through Head Mounted Displays (HMDs) that present immersive content for the viewer. Empirical studies have begun exploring how immersive content delivered through HMDs can be used to support learning in professional healthcare training, for example providing students with engaging learning experiences where they can interact with clients presenting with different symptoms and diagnosis without jeopardizing client safety (Mavrogiorgou et al., 2022; Mantovani et al., 2003). Such immersive experiences can help students become more aware of their own emotional and cognitive responses during clinical interactions, with the potential of fostering greater self-knowledge. Studies have also demonstrated that VR-based training has a small effect on improved knowledge retention compared to traditional methods among health-care students (Woon et al., 2021).

In conclusion, VR offers a promising and innovative approach to training clinical psychology students, allowing them to practice key skills in a highly immersive, controlled, low-risk environment. It is for example well known that VR-simulations are emotionally evocative (Chirico and Gaggioli, 2019). By simulating emotionally charged scenarios, VR could allow students to practice managing intense emotions and reflect on their internal responses in a safe learning environment. This might provide students with learning experiences that may facilitate self-knowledge and moment-to-moment self-awareness, both considered critical components of clinical practice that enable clinicians to manage their emotional responses and attune to clients. However, when using VR in educational settings, it is important to be mindful of symptoms of discomfort and motion sickness reported by some users. A meta-analysis by Saredakis et al. (2020) found that disorientation, oculomotor issues and nausea were the most commonly reported symptoms across multiple VR studies. It is therefore essential to investigate how motion sickness might also impact on the experience and learning opportunities provided by VR in clinical training.

### Aim

The purpose of this proof-of-concept article is to present an example of how 360-videos administrated through Head Mounted Displays (HMD) can be integrated in clinical training to give students added learning experiences that give opportunities for strengthened self-knowledge and moment-to-moment self-awareness (conceptual contribution) and explore students' experience of such learning activities (empirical exploration).

## Methods

### Development of stimulus material

The VR exercise was developed in the context of a 6-year integrated program in clinical psychology. The first author, who leads a first-year course with learning outcomes related to recognizing emotional expressions and reflecting on personal development relevant to clinical work, found it challenging to provide sufficient learning opportunities for these goals in large student groups (around 100 students). To address this, she designed a VR exercise that combined emotion recognition training with opportunities to develop self-knowledge and moment-to-moment self-awareness. Based on these learning outcomes, stimulus material was made with the support of a professional photographer from UiB Learning Lab. Eight professional actors were asked to display non-verbal expressions of one emotion each. The actors were seated in a therapy room and were filmed with a 360-camera from the position where a therapist normally would be seated. The sound was recorded with a separate microphone, and the sound recording was synchronized with the film to make the stimuli as immersive as possible. The first author and the photographer then cut the films to make stimuli lasting 60–90 s per emotion. For an overview of the order and length of the stimuli, see Table 1. We assessed that the contrast between the high-quality video of actors displaying authentic emotional expressions and the animated quality of a virtual therapist body being placed where the 360-camera was standing would decrease sense of presence. Students were therefore not embodied in the stimulus material in ways of seeing their own hands or a virtual therapist body.

**Table 1:** Sequence and duration of stimuli.

**Clip number**	**Emotion**	**Gender of actor/actress**	**Duration**
1	Sadness	Male	94 s
2	Contempt	Female	80 s
3	Fear	Female	68 s
4	Cold anger	Male	69 s
5	Neutral/silence	Female	82 s
6	Shame	Male	94 s
7	Hot anger	Female	60 s
8	Warmth/joy	Female	82 s

### Hardware and software description

The hardware used for Head Mounted Displays (HMDs) was Pico Standalone Headsets, displaying stimuli at a resolution of 1,920 × 2,160 per eye with a 75 Hz refresh rate. The headsets provided a 101° diagonal field of view and supported an interpupillary distance range of 54–71 mm. Audio was delivered through Sennheiser HD 25 (70 Ω) headphones connected to the HMDs via a 3.5 mm cable. The HMDs were remotely controlled using Showtime VR software (version 4) installed on a Dell Latitude 7400 laptop (Intel Core i5 CPU). A dedicated 5 GHz Wi-Fi Router was configured to operate on a closed network without internet access to ensure secure and stable connection between the Showtime VR software and the HMDs. The laptop and router were placed within an 8-m radius of the HMD workstations to ensure a stable wireless connection.

### Procedure during the VR-exercise

Participation in the VR exercise was part of ordinary clinical training and was mandatory. 94 students were enrolled, and students completed the VR exercise in established groups, with 18–20 students in each group. In the classroom, we had set up 10 workstations on desks with 10 HMDs and headsets, together with a sheet of paper with the exercise and instructions. Students were seated in comfortable chairs in front of the desk. There was ~1–2 m of space between each workstation. The two authors instructed students throughout the exercise. Two instructors were present during the exercise to manage any potential cases of nausea, motion sickness, or overwhelming emotional reactions; however, no such incidents occurred.

Students were instructed to observe and reflect on their own reactions as well as what they believed the other person experienced while watching each of the eight clips. Following each clip, students were asked to put the HMDs on their forehead and to write down *1) What happened in you as you watched the clip?; 2) What emotion do you think the other person was experiencing?; 3) What sources of information did you use to decide what the person in the film was feeling?* Then they were instructed to put on their HMDs again, and the next clip was played. The same sequence of 360-degree video clips was used in all student groups (i.e., Table 1). The students were also asked to include their experiences from this VR exercise in an essay relating to the overarching focus of the course (communication and relational skills).

### Proof-of-concept study

#### Questionnaires

Background variables: Questions mapping participants' gender and age and prior experience with using HMDs (see Supplementary material for survey questions developed for this study).

Immersion and presence: The Slater-Suoh-Steed Questionnaire (SUS; Usoh et al., 2000) was used to assess the degree of immersion and presence during the VR exercise. The SUS consist of six items rated on a 7-point Likert scale ranging from 1 (to a very small extent) to 7 (to a very large extent), with items such as “To what extent did you feel that the simulated situation was real?”. Internal consistency was good in the present sample (Cronbach's alpha = 0.86). Higher scores indicate higher immersion and presence.

Simulation sickness: The Simulation Sickness Questionnaire (SSQ; Kennedy et al., 1993) was used to assess the degree of motion or simulator sickness experienced during the exercise. Participants rated 16 symptoms (e.g., nausea, disorientation, eyestrain, dizziness and headache) on a 4-point Likert scale ranging from 1 (none) to 4 (severe). Internal consistency was acceptable in the present sample (Cronbach's alpha = 0.67). Higher scores reflect higher simulation sickness.

Emotion recognition in self and others: We developed questions to measure how effectively participants felt they could identify their own emotional responses, both more generally and concretely during the VR exercise. These items were rated on 5-point Likert scale from 1 (very difficult) to 5 (very easy).

Self-knowledge and self-awareness: We developed questions to tap whether participants discovered something new about themselves and their reactions in emotional situations during the VR exercise, as well as participants' perceived impact of the VR exercise on improving their ability to recognize emotions in themselves and others.

#### Recruitment and consent

A total of 94 students were enrolled in the course that included the VR exercise. Because the VR exercise was a mandatory part of the clinical training, the internal review board and data protection officer instructed us to make sure there was a clear distinction between the VR exercise as a learning activity and this proof-of-concept study. Invitation to participate in this study was therefore sent to all students 5 days after the VR exercise. One reminder was sent after 1 week. Data collection closed after 3 weeks. Completion of the survey was regarded as consent to participate.

#### Analyses

All quantitative data were analyzed using Python version 3.9.19, with the modules pandas, numpy and scipy. Descriptive statistics (means and standard deviations) for numerical variables were computed, and the data were categorized and labeled according to age, gender, and previous VR experience using predefined mappings.

Group comparisons between males and females were conducted using an independent samples *t*-test. Similar group comparisons were conducted between participants with no experience and those with prior VR experience. Pearson correlation coefficients were calculated to explore the relationships between selected variables, including emotional recognition, SUS, and SSQ scores. Correlation matrices were created and visualized using heatmaps, with *p*-values annotated for each pairwise correlation.

Qualitative data was coded line-by-line by the first author, using NVivo (Lumivero, 2023) as technical support. Codes were then grouped together, using principles from analysis of patterns across participants (Braun et al., 2019). The qualitative results were then actively compared to the quantitative results, to utilize the access to both qualitative and quantitative data.

#### Approval of the proof-of-concept study

In line with regulations, the local data protection officer was consulted to decide the appropriate body for assessing and approving the study. Because the study did not include health information, the study was assessed, approved, and registered in the University of Bergen's system for risk and compliance, RETTE (reference R3320).

## Results

42 students consented to participate in the proof-of-concept study. Of these, one participant did not fill out the questionnaire SSQ, and was therefore excluded from the analyses conducted with that instrument. Participant characteristics are presented in Table 2.

**Table 2:** Participant characteristics.

	***n***	**%**
**Age**		
18–20	13	31
21–24	24	57
25–29	5	12
**Gender**		
Female	35	83
Male	7	17
**Previous experience with VR**		
None	17	40
Limited	25	60

Reflecting the gender imbalance among students in our training context, the sample consisted of 35 females and 7 males. All males had some limited usage of HDMs, while half of the females had never used HMDs before (*n* = 17). The students found it easier to recognize own emotions in general (M = 3.38; SD = 1.03) than when they were doing the VR-exercise (M = 2.98; SD = 0.98). The mean total of SUS, SSQ and emotion recognition indices are summarized in Table 3.

**Table 3:** Participant scores SSQ, SUS and emotion recognition.

	**M**	**SD**
**Sense of presence and simulation sickness**		
SUS	20,12	6,88
SSQ	19,10	3,18
**Self-perceived emotion recognition capacity in general**		
	M	SD
Others	3,93	0,64
Self	3,38	1,03
**Self-perceived emotion recognition capacity in VR exercise**		
	M	SD
Others	3,60	0,73
Self	2,98	0,98

SUS, slater-suoh-steed questionnaire; SSQ, simulation sickness questionnaire.

### Students' experiences of the VR exercise

The qualitative data showed that participants generally experienced the task of recognizing emotions in others as manageable: “It went well. Even though you are very aware that you are in a simulated context, the experiences in the HMDs are sufficiently realistic, so that the task of capturing the other's emotions was not too hard” (participant 108). Some participants found, however, that the degree of difficulty varied between clips and that the lack of situational information in relation to the stimuli made it difficult to interpret what the other was feeling: “It wasn't necessarily difficult to capture that there were different emotions in play. But it was difficult to pin-point the emotion because I did not know anything about the context or the background” (participant 129).

Many participants also found it easier to recognize emotions in others compared to recognizing one's own emotions: “I am less familiar with focusing on what is happening in me, while I focus on someone else's body language. Yet, it feels important. Especially when I feel directly affected by (the other) by feeling threatened or uncomfortable” (participant 130). This was also reflected in the quantitative data. 76.2% reported that they generally found it quite or very easy to detect what emotions others were feeling. The corresponding number regarding one's own emotions was 47.6%. Self-reported ease of detecting emotions during the VR exercise showed the same pattern: 54.7% found it quite or very easy to detect what emotions the people in the films were feeling, while 33.4% found it quite or very easy to detect their own reactions during the VR exercise.

The difference between detection of emotions in others vs. oneself was also reflected in the perceived benefit of the VR exercise. 33.4% of the students experienced that the VR exercise to a large or very large degree strengthened their ability to recognize emotions in others. The corresponding number for perceived ability to recognize emotions in oneself was 38.1%. About a quarter of the students did not experience that the VR-exercise strengthened their emotion-recognition skills significantly (23.8% for emotions in others, 26.2% for one's own emotions). Correlation analyses revealed that the extent to which participants felt the VR exercise improved their ability to recognize both their own and others‘ reactions was significantly correlated with their sense of presence in the VR situation (*r* = 0.47, *p* = 0.002 for self-recognition; *r* = 0.44, *p* = 0.003 for recognizing others). That is, the more the students felt immersed and present in the VR situation, the more they found the VR exercise to improve their ability to recognize their own and others' affective reactions.

The participants' evaluation of whether the VR-exercise improved their ability to detect their own or others' emotions did not correlate with how easy they generally found it to recognize own or others' emotions (r's = 0.01–0.2; p's <0.211). However, participants who generally found it easier to recognize others' emotions also tended to find it easier to recognize their own emotions, both generally (*r* = 0.37, *p* = 0.015) and during the VR exercise (*r* = 0.31, *p* = 0.046). Additionally, there was a strong correlation between the perceived ease of recognizing others' emotions more generally and perceived ease in identifying the emotions of the people in the films (*r* = 0.66, *p* < 0.001). Similarly, the perceived ease of recognizing one's own emotions more generally correlated with perceived ease of recognizing both the emotions of the people in the films (*r* = 0.34, *p* = 0.030) and one's own emotional responses during the VR exercise (*r* = 0.44, *p* = 0.003).

Self-reported sense of presence correlated negatively with self-reported overall ease with detecting how other people feel (*r* = −0.35, *p* = 0.022). That is, the easier students normally found it to detect how other people feel, the less presence in the VR-situation was reported. In relation to this, the qualitative data showed that many participants experienced that the situation of the VR exercise, watching simulated material with actors, and sitting with other students, muted their own reactions, thus increasing the difficulties of recognizing their own reactions: “On my part I became very aware it was acting, so I did not get so strong reactions, but I definitely felt the ambience of the films more strongly than I would have on a screen!” (participant 84). However, some participants were surprised about how real the situation felt: “I really experienced that the persons for example looked into my eyes, something I did not expect at all. I think there is quite a big difference from watching a regular video” (participant 94), and how much information was available in non-verbal behavior: “How much knowledge you can get just based on body language” (participant 128).

### Group differences

Although results should be interpreted with caution due to small groups and a large gender imbalance, analysis showed that females reported greater overall discomfort with VR than males. This was indicated by higher total scores on the Simulation Sickness Questionnaire (M = 19.40, SD = 3.32) compared to males (M = 17.33, SD = 1.21; T = −2.76, *p* = 0.012). When investigating each item of the SSQ individually, it was only item number 1 (“General discomfort”) that showed statistically significant differences between females (M = 1.49, SD = 0.56) and males (M = 1, SD = 0; T = −5.11, *p* < 0.001). Moreover, when testing whether experience with HMDs influenced overall discomfort, *t*-tests showed no statistically significant difference between participants with no experience (M = 19.59, SD = 3.30) and limited experience with VR-goggles (M = 18.75, SD = 3.11; T = −0.82, *p* = 0.418).

In terms of presence achieved in the exercise there was no difference between males and females (T = −0.367, *p* = 0.72) nor between groups with and without prior experience with using HMDs (T = 1.47, *p* = 0.149).

## Discussion

The present proof-of-concept study aimed to explore the use of virtual reality (VR) and 360-degree video clips as a tool for enhancing clinical psychology students' self-knowledge and moment-to-moment self-awareness – both conceptually and empirically. We provided psychology students novel learning opportunities and systematized their experiences with VR and with immersive stimuli where professional actors engaged in non-verbal emotional expressions. The VR exercise represents an example of how new technology can be used to create new learning activities for students that facilitate therapist development. Our results show promise for integrating VR simulations into clinical training to provide immersive, controlled environments where students can practice key clinical skills, including emotional recognition in themselves and others. They also point to important challenges to address in the future.

Although the ability to know and understand oneself and to be aware of how one is affected and reacting in a particular moment is considered vital in clinical work (e.g., American Psychological Association, 1947, 2006; Benbassat and Baumal, 2005; Jennings and Skovholt, 1999; Rodolfa et al., 2005), there have not been established ways to measure self-knowledge and moment-to-moment self-awareness, nor agreed ways to integrate such a focus in clinical training. For example, both a broader focus on personal development and a narrower focus on self-awareness training have been integrated in clinical training (Grimmer and Tribe, 2001; Knapp et al., 2017a,b; Luke and Kiweewa, 2010; Payne, 1999; Topuz and Arasan, 2014). While considered useful learning experiences for students, ethical concerns have been raised regarding making personal therapy and experiential groups mandatory (Atkinson, 2006). Moreover, differentiating direct from more indirect approaches to facilitate students' self-knowledge, Benbassat and Baumal (2005) has pointed to challenges in both types of approaches, for example the risk of direct approaches to leave the students in the position of passive observers. It is therefore interesting to explore the potential of 360 degree videos and HMDs as a low-risk learning tool that engages students in activities that might provide opportunities for enhanced self-knowledge and possibilities to practice moment-to-moment self-awareness.

We aimed to maximize students' learning opportunities for self-knowledge moment-to-moment self-awareness by having them take notes after each clip regarding their own reactions and which sources of information they relied on when deciding which emotions the people in the films experienced. They were then asked to reflect on their experiences from the VR exercise and include these reflections in an essay, helping them consolidate their insights from the experiential activity. Although great care went into the construction of the VR exercise our results both point to the need for further research and pedagogical development to refine how VR can be integrated into clinical training to provide a safe training arena for such skills. For example, self-reported ability to recognize emotions in oneself and others was not correlated with perceived benefit of the VR exercise. This is important, as future efforts should focus on how to strengthen students who experience that they lack key therapeutic competencies. It is also interesting to note that students reported that they normally found it easier to recognize emotions in themselves and others than they did during the VR-exercise. This might point to challenges in providing a fully immersive environment with non-verbal stimuli, as the qualitative data showed that the lack of context made it difficult to interpret the emotional expression. At the same time qualitative data showed how the VR-experience also made students reflect on and become aware of the amount of information available in non-verbal communication. This result might therefore also point to difficulties with how students interpreted the wording of the questions.

The benefits and challenges with different types of stimulus material for supporting self-knowledge and self-awareness are important to explore in future research. An important finding from this proof-of-concept study is, however, the significant role that immersion and presence played in the perceived benefits of the VR exercise. Participants who reported a stronger sense of presence in the VR simulations also rated the exercise as being more beneficial in improving their ability to recognize both their own and others' emotions. This aligns with previous research suggesting that the sense of presence, that is, feeling as though one is truly “there” in a virtual environment, enhances the emotional impact of VR simulations (Woon et al., 2021). The fact that some participants felt the simulated experience was so realistic that they could make meaningful interpretations of others' emotions despite knowing it was a simulation, underscores the potential of VR to replicate real-life social interactions in a controlled environment. As previous studies have shown, VR can evoke strong emotional responses, even in scenarios where participants know the context is artificial (Mantovani et al., 2003). This immersion allows for deeper engagement, enabling students to practice emotional recognition and self-awareness in ways that traditional classroom methods or videos may not facilitate.

Although presence was correlated with perceived benefit of the VR exercise, there was a negative correlation between sense of presence in the VR exercise and self-reported emotion-recognition ability. One interpretation of this result is that people that self-report as more skilled in recognizing emotions in others are more easily disturbed by the artificial qualities of the simulated material and situation, and that this makes it more difficult to feel as if they are in a real situation. This might have important implications for which learning activities should be offered which students and need to be explored further in future research. Either way, the results point to the importance of paying attention to content quality if we are to maximizing the benefits of technology-enhanced learning, including creating realistic, emotionally engaging stimuli. This finding also highlights the importance of the properties of simulations that enhance their realism and immersion. Future research should therefore explore the differential effect of 360-video with use of professional actors and carefully crafted scenarios and animated avatars in heightening the sense of presence in different groups of students.

It is also important to note that both qualitative and quantitative data showed that many participants found it easier to recognize others' emotions than their own emotions. This has implications for training given the significance of moment-to-moment self-awareness in clinical practice (e.g., American Psychological Association, 1947, 2006; Benbassat and Baumal, 2005; Jennings and Skovholt, 1999; Rodolfa et al., 2005; Watson and Wiseman, 2021). Interestingly, self-reported ability to recognize emotions in others correlated positively with self-reported ability to recognize own emotions. This points to the importance of providing safe learning arenas where students can practice a double focus on what is happening in the other person while noticing their own reactions—but without having to respond to or take care of the other person—and how VR can provide unique opportunities in this respect (Mantovani et al., 2003). It also points to the importance of future research exploring the causal direction of these relationships, and whether an increased ability to recognize others' emotions also increases students' capacity for moment-to-moment self-awareness.

Our results also point to the importance of paying attention to contextual effects of the classroom environment on learning and demonstrate the important distinction between helpful and hindering self-awareness (Williams et al., 2003, 2008). For example, qualitative data showed that the presence of other students in the classroom elicited hindering self-awareness in some students, that reduced opportunities for experiential learning during the VR exercise. Others experienced that their own reactions were muted in this setting—both due to others being present in the room, but also because they knew the people in the films were actors. This experienced muting of emotions during the VR exercise was also reflected in the quantitative data. Students who reported this still felt that their reactions elicited in the VR exercise were stronger than they would have been if the simulated material was only shown on a screen. The use of head mounted devices to facilitate immersive learning can thus both elicit insecurity in students because they cannot see the other students in the classroom while doing the exercise. At the same time, it is the exact same mechanism of being separated from what is happening around you that makes it possible to immerse yourself in the stimuli, thus engage in active, experiential learning. These tensions and dilemma point to the careful thought that needs to go into the design of such learning activities if they are to maximize students' learning opportunities. Features such as layout of the classroom, set-up of the stations with the HMDs, group size, etc., should be carefully considered. From our experience with this specific exercise, student groups larger than 20 students would not be recommended, even with two instructors. Ideally the number of head mounted devices should match the number of students. Future research should explore the effects of physical learning environment and organization of learning activities using HMDs more systematically.

Finally, although the results should be interpreted with care given the small group and large gender imbalance, it is important to note that females in our sample reported higher degrees of simulation sickness compared to males. This finding contrasts with some existing research, which suggests that there are no significant gender differences in motion sickness or simulation sickness susceptibility (Saredakis et al., 2020). Our results may point to other factors than gender being at play, such as differences in prior experience with VR or individual variability in sensory sensitivity. In our study, all males had some prior experience with HMDs, while this was true for only half of the female participants. Familiarity with VR could have contributed to a reduced discomfort. Nevertheless, the lack of a statistically significant correlation between prior VR experience and discomfort suggests that other individual differences, such as motion sickness susceptibility could also contribute to these gendered experiences (Chang et al., 2020). Given the mixed findings in the literature regarding gender differences in VR experiences, future studies should aim to explore these differences further. Either way it is crucial to ensure that VR training environments are accessible and comfortable for all participants, particularly if integrating the use of VR into clinical training.

## Limitations and methodological reflections

The present article reports on conceptual and empirical work to begin exploring whether 360-degree-videos and HMDs have potential in facilitating the development of self-knowledge and moment-to-moment self-awareness among psychology students. Although providing a concrete example of how VR can be used to expand on learning experiences provided in clinical training and explores students' experiences of this type of exercise, the study sample is relatively small, with less than 50% of students participating in the study. There is therefore a risk of self-selection bias, with students more comfortable with technology or less prone to motion sickness being more likely to participate. The sample was also, in line with the gender composition of our students, skewed with a clear overweight of female participants. Reflecting the stage of exploration, the simulated material used in this proof-of-concept study were not standardized to the level that would be required in an experimental design. The different clips ranged from 60 to 94 s, and more clips included a female actor. These represent serious limitations in relation to generalization of the results. It is also important to note that we only collected self-report data on perceived benefits of the VR-exercise, rather than scoring accuracy in emotional recognition or measuring observer-rated performance in other ways. The possibility of including performance-based measures as well as higher degree of standardization of stimuli is important to consider in future research.

It is also important to note that both authors share enthusiasm for the potential in including HMDs in clinical training to provide students with more immersive experiences than is possible in roleplay, while representing a safe arena for trial and error without the possibility of inflicting harm on patients. The first author had already very positive experiences from using HMDs with 360-degree movies with professional actors in a course on trauma assessment in the 4^th^ year of training. Both authors also had extensive and positive experience with producing realistic, simulated material for training purposes with high potential for immersion and presence by using professional actors and 360-camera. Given the context of training students in clinical psychology, both authors consider the largest potential in such learning activities to be opportunities for students to increase self-knowledge and self-reflection capacity by noticing their own reactions and action tendencies in different clinical situations, as well as opportunities for practicing heightened moment-to-moment self-awareness and regulation of own activation. In this context we do therefore not consider VR as a tool to teach students specific responses in specific situations. We believe trainers' enthusiasm is important for successful implementation of technology-mediated clinical skill training, but this starting point might also have implications for the transferability of the results to new training contexts.

## Conclusion

In conclusion, this study provides a concrete example of how VR simulations can be integrated into clinical training to facilitate self-knowledge and moment-to-moment self-awareness in clinical psychology students. The VR exercise was generally well received by the students, and the study shows that integration of this type of learning experiences where students are given opportunities to engage deeply with emotionally evocative scenarios in controlled and safe learning environment as part of clinical training is feasible. Students generally found it easier to recognize others' emotions compared to their own. Perceived benefit of the VR exercise was not related to self-reported ease of emotion recognition. Rather sense of presence in the VR exercise was correlated to perceived benefits. This highlights the importance of high-quality, realistic simulations in clinical training, and paying attention to potential hindrances for learning, such as simulation sickness, physical learning environment, and task administration, when constructing such learning experiences. There is need for future research and pedagogical development to support the integration of technology-enhanced learning using head mounted displays and 360-degree videos in the best possible way.

## Data Availability

The raw data supporting the conclusions of this article will be made available by the authors, without undue reservation.
