# Supplementary material for: Virtual reality as a tool to enhance clinical psychology students' self-knowledge and self-awareness—a proof-of-concept study
Source: Front Psychol. 2026 Jan 5;16:1659873. doi: 10.3389/fpsyg.2025.1659873 (PMC12813128; doi:10.3389/fpsyg.2025.1659873)
Supplement: Supplementary file 1 [file Data_Sheet_1.pdf]

Supplementary material:

Survey Questions developed for the proof-of-concept study:

1. **How old are you?** (18–20; 20–25; 25–30; 30–35; 35–40; 40+)
2. **Gender** (male/female/other)
3. **How familiar are you with using VR headsets?** (first time; used a little before; used quite a bit; used a lot)
4. **How easy do you usually find it to recognize what emotions others are experiencing?** (Likert scale 1–5; very difficult – very easy)
5. **How easy do you usually find it to identify what you yourself feel in different situations?** (Likert scale 1–5; very difficult – very easy)
6. **How easy did you find it to recognize what the people in the film were feeling?** (Likert scale 1–5; very difficult – very easy)
7. **Please elaborate (open question)**
8. **How easy was it to notice what was happening within yourself while watching the films?** (Likert scale 1–5; very difficult – very easy)
9. **Please elaborate (open question)**
10. **Was there anything that surprised you during the VR exercise?** (open question)
11. **Did you discover anything new about yourself and your reactions during the VR exercise? Please elaborate** (open question)
12. **How was it to apply experiences from the VR exercise in writing the essay?** (open question)
13. **To what extent do you feel that the VR exercise improved your ability to recognize others' emotions?** (Likert scale 1–5; very small degree – very large degree)
14. **To what extent do you feel that the VR exercise improved your ability to notice your own reactions?** (Likert scale 1–5; very small degree – very large degree)
